# P-2051. Clean Teeth and Oral Airway: A Strategy to Reduce IVAC Plus SIR

**DOI:** 10.1093/ofid/ofaf695.2215

**Published:** 2026-01-11

**Authors:** Tomoko Ray

**Affiliations:** AdventHealth Orlando, Apopka, FL

## Abstract

**Background:**

Intensive Care Unit faced an increase in Infection-related Ventilator-Associated Complication (IVAC) and Possible Ventilator-Associated Pneumonia (PVAP) in 2023. These VAEs can lead to longer lengths of stay and increased mortality. Despite oral care every two hours and toothbrushing every shift being the current practice in our unit, compliance was low. Therefore, the unit leaders and infection preventionist recognized an opportunity for improvement in the current protocol, which is an evidence-based practice strategy for VAE prevention.

**Methods:**

In June 2023, unit leaders implemented a new education campaign based on best practice recommendations, and focused on oral care and toothbrushing to prevent VAEs, the proper technique, and documentation.

The infection preventionist reviewed documentation daily to provide feedback to further educate staff.

These leaders rounded on ventilated patients to check their teeth cleanliness daily. Despite initial implementation, IVAC and PVAP counts remained high. In September 2023, the infection preventionist identified that endotracheal tubes (ETTs) were developing white biofilms. Consequently, education was updated with ETT brushing on October 1, 2023.

To compare the effectiveness of this oral care project in preventing VAEs, IVAC Plus (IVAC and PVAP) standardized infection ratios (SIRs) from the National Healthcare Safety Network (NHSN) were utilized. The NHSN statistical calculator was used to analyze the significance of the results (p-value).

**Results:**

The IVAC Plus SIR was 3.018 before the full implementation of the education and compliance project (January 2021 to September 2023). It was reduced to 2.455 by 18.65% post-implementation (October 2023 to February 2025), although it was not a significant reduction yet: [p-value = 0.4764 (95% Confidence Interval = 0.456, 1.41) using the NHSN statistical calculator]. The trend is going down.

**Conclusion:**

A coordinated effort by unit leaders and infection prevention, involving staff education and compliance monitoring with daily feedback, is important for maintaining VAE reduction. Any facilities struggling to reduce NHSN reportable VAE events should pay close attention to oral care every two hours and teeth and endotracheal tube brushing every shift to reduce their IVAC Plus SIR.

**Disclosures:**

All Authors: No reported disclosures

